# Hospital costs associated with post-traumatic stress disorder in somatic patients: a retrospective study

**DOI:** 10.1186/s13561-020-00281-0

**Published:** 2020-07-11

**Authors:** Rieka von der Warth, Philip Hehn, Jan Wolff, Klaus Kaier

**Affiliations:** 1 Section of Health Care Research and Rehabilitation Research, Medical Center – University of Freiburg, Faculty of Medicine, University of Freiburg, Hugstetter Str. 49, 79106 Freiburg, Germany; 2 Institute of Medical Biometry and Statistics, Faculty of Medicine and Medical Center – University of Freiburg, Freiburg, Germany; 3 Department of Psychiatry and Psychotherapy, Medical Center - University of Freiburg, Faculty of Medicine, University of Freiburg, Freiburg, Germany

**Keywords:** Post-traumatic stress disorder, Hospital costs, Comorbidity, Cost-of-illness, Diagnosis-related groups

## Abstract

**Background:**

Post-traumatic stress disorder is likely to affect clinical courses in the somatic hospital ward when appearing as comorbidity. Thus, this study aimed to assess the costs associated with comorbid post-traumatic stress disorder in a somatic hospital and to analyze if reimbursement appropriately compensated additional costs.

**Methods:**

The study used data from a German university hospital between 2011 and 2014, analyzing 198,819 inpatient episodes. Inpatient’s episodes were included for analysis if they had a somatic primary diagnosis and a secondary diagnosis of post-traumatic stress disorder. Costs were calculated based on resource use and compared to reimbursement. Analyses were adjusted for sex, age and somatic comorbidities.

**Results:**

*N* = 219 Inpatient’s episode were found with primary somatic disorder and a comorbid post-traumatic stress disorder. Inpatients episodes with comorbid post-traumatic stress disorder were compared to 34,229 control episodes, which were hospitalized with the same main diagnosis. Post-traumatic stress disorder was associated with additional hospital costs of €2311 [95%CI €1268 - €3355], while reimbursement rose by €1387 [€563 - €2212]. Results indicate that extra costs associated with post-traumatic stress disorder are not fully reimbursed. Male patients showed higher hospital costs associated with post-traumatic stress disorder. On average, post-traumatic stress disorder was associated with an extra length of stay of 3.4 days [2.1–4.6 days].

**Conclusion:**

Costs associated with post-traumatic stress disorder were substantial and exceeded reimbursement, indicating an inadequate reimbursement for somatic patients with comorbid post-traumatic stress disorder.

## Introduction

Post-traumatic stress disorder (PTSD) is diagnosed in individuals who were exposed to a traumatic event or who witnessed a traumatic event, when the following symptoms are observable: high physical arousal, negative thoughts leading to avoidance of trauma-related stimuli, and intrusive symptoms such as flashbacks or nightmares [DSM-V]. Individuals with PSTD have previously been shown to suffer from severely impaired health-related quality of life and struggle with a decreased life satisfaction [1, 2]. PTSD was previously found to have a lifetime prevalence of 1% to 8% [3–5] with women having a higher prevalence than men [3].

There is some evidence that psychiatric comorbidities are associated with additional hospital costs. A meta-analysis found depression in particular to be associated with significantly higher hospital costs [6]. In patients with back pain undergoing disc surgery, the presence of a comorbid psychiatric disorders predicted higher direct and indirect health care costs. Associated costs were found even though almost no mental health services were utilized by the patients [7]. In a retrospective study assessing the associated costs of psychiatric comorbidities additional costs of 1344€ were found [8]. Moreover, in a study assessing the associated extra costs of mental disorders in an internal and psychosomatic department, reaction to severe stress, and adjustment disorders were found to have associated costs of 191€ per inpatient [9].

PTSD as main diagnosis was associated with annual direct excess health care costs ranging from 512 US-$ to 19,435 US-$ per annum in a recent systematic review assessing the costs of illness [10]. Higher health care costs in patients with PTSD are observable across a variety of settings. Costs of patients with PTSD exceed the costs of patients without PTSD by between 8% and 75% when PTSD occurred as a result of traffic accidents [11, 12], and there is some evidence that poor psychological health and psychological disorders, such as PTSD, complicate clinical course even in routine surgical procedures [13]. Yet there are dissenting findings how PTSD affects the clinical course [14]. In a study assessing patients admitted to a Level I Trauma Centre, post-traumatic stress syndrome (PTSS) following traumatic injury was associated with an average length of stay (LOS) increase of 2 days [15], as poor psychological health in general was associated with an increased LOS in several studies [16, 17].

An increase in LOS is especially significant since LOS is not taken into account in the German Diagnosis Related Groups (G-DRG)-based reimbursement system in full proportion to the additional days of stay. DRGs are the most common hospital reimbursement system internationally, implemented in Germany in 2004. DRGs aim to create homogenous groups of patients on national level and are yearly reviewed, by approximately 16% of all hospitals in Germany providing case-cost data, clinical case data (e.g. diagnosis, procedures and sociodemographic) and additional service data on patient level [18, 19]. Within each patient group, criteria such as diagnosis, age, gender and others are then used to define financial reimbursement [20]. However, if financial reimbursement does not properly reflect costs, service providers might try to reduce costs at the expense of treatment quality [21].

To our knowledge there is no study assessing the associated hospital costs or adequate reimbursement of only PTSD as comorbid disorder in somatic patients. A previous study found additional reimbursement associated with psychiatric comorbidities in general, however, the additional reimbursement under the current G-DRG system did not cover the higher costs [8]. Thus, an inadequate reimbursement of PTSD can be hypothesized.

Cost of illness studies (COIs) display the economic costs of an illness, providing scientific evidence for political decision makers in order to take further actions, such as treatment or research [22, 23]. Thus, knowing the costs of illness of a disorder with all influencing factors, such as the impact of comorbidities, is important to improve the health status or patients and rationalise costs due to efficiency [24]. There are different methods available for COIs, but as modelled studies are based on assumptions, some authors ask for more studies alongside clinical studies or at least within the clinical setting [25]. Thus, this study aimed to determine the impact of PTSD on in-hospital costs of patients hospitalized due to somatic diseases. Furthermore, we determine how well the G-DRG system compensates its cost. By this, this study will not only inform political decision makers on the health care costs of comorbid PTSD in somatic patients, but will also provide first information for researchers to develop new research questions on new treatments of PTSD or in-hospital routines.

## Methods

Data collection was conducted at a university hospital in Germany between 2011 and 2014. Inpatients episodes were included in this study if they were hospitalized for somatic reasons and had a documented PTSD as a comorbid disorder. Presence of comorbid PTSD was defined as the presence of the International Statistical Classification of Diseases and Related Health Problems, 10th revision, German modification (ICD-10-GM) code for PTSD (ICD-10 F43.1) as secondary diagnosis. Only adult patients aged > = 18 years were included. Inpatients episodes with a psychiatric main diagnosis were excluded. All inpatient episodes between 2011 and 2014 with an minimum age of 18 year and that had no documented comorbid psychiatric disorder were considered as potential controls (non-PTSD). Stratification by the ICD-10 main diagnosis code was applied to ensure comparability based on diagnostic and therapeutic regimes. As a result, only non-PTSD control episodes which were hospitalized with the same main diagnosis as PTSD inpatient episodes were considered for the analysis.

Resource use was obtained from the hospital’s department of management. Using a bottom-up micro costing approach, full operating costs were calculated per inpatient episode according to the standardized costing system using actual costs per case occurred in conformity with the calculations of the German Institute for Hospital Reimbursement (InEK) [18]. Cost categories included are staff costs for physicians and nursing, material costs and infrastructure costs [18]. Within the three cost categories, 11 different cost centres are then calculated, generating an individual cost-matrix per episode [26]. Costs calculated are based on real costs using a full cost approach. Direct costs are based on the documented utilization using unit costs, whereas overhead costs and costs on primary costs units are based upon resource use such as, time in operating room or laboratory [26, 27]. Capital costs were excluded as those are not reimbursed by the G-DRGs. Thus, a health care system perspective was applied. Furthermore, the standardized InEK cost accounting scheme allows the calculation of reimbursement for each episode, which was used to assess reimbursement in this study. A detailed overview of the cost accounting scheme can be found in the cost calculation manual by the InEK [28].

Costs and reimbursements were valued and adjusted to 2014 €. Data included main and secondary diagnoses, age, sex, LOS, discharge status and intensive care hours. Main diagnoses based on the 4-digit ICD-10 were included as categorical fixed effects, while age and sex were included as potential confounders. Furthermore, the Charlson comorbidity (CCI) index was calculated and included into the analyses as a continuous covariate, to control for severe comorbidities.

As primary endpoints we used in-hospital costs per episode calculated according to the standardized InEK costing system, as described above [18, 29]. As secondary endpoints, reimbursement and LOS per episode were analyzed analogously. Additional analyses were carried out in order to identify whether sex, age or the CCI, act as affect modifiers regarding the impact of PTSD on the primary and secondary endpoints. Therefore, their impact was tested using interaction terms. We assume that hospital costs, reimbursements and LOS were non-normally distributed and right-skewed and therefore used a generalized-linear model (GLM) with a log link and a gamma distribution for all regression analyses. We detected no outliers and no missing data. Thus, a GLM based on quasi-likelihood estimators was deemed appropriate. All analyses were conducted using Stata 15.1 (StataCorp, College Station, TX, USA).

## Results

As no official checklist for COIs exits, results reported in this study follow the Consolidated Health Economic Evaluation Reporting Standards (CHEERS) where applicable [30].

In total 198,819 inpatient episodes were recorded between 2011 and 2014, of whom *N* = 219 were found to be patients with a primary somatic disorder and a comorbid PTSD. *N* = 34,229 inpatient episodes with the same main diagnosis as those with PTSD were found. Thus, as shown in Table 1, 219 PTSD inpatient episodes were compared to 34,229 non-PTSD inpatient episodes. PTSD inpatient episode were found to be more often female patients (*p* < 0.001), younger patients (p < 0.001), and had less severe comorbidities as indicated by the CCI (p < 0.001). PTSD was associated with longer length of hospital stay. Mean costs per inpatient episode of €7919 in patients with comorbid PTSD were found, whereas patients without comorbid PTSD showed mean costs per inpatient episode of €5253. Male patients with comorbid PTSD showed higher mean costs of €11,021 per episode. Reimbursement was lower than costs per episode in patients with comorbid PTSD, with an average reimbursement of €7081 per episode. In contrast, a positive net reimbursement was observed in patients without comorbid PTSD.

**Table 1:** Descriptive statistics of inpatients episodes included in the analysis

	All PTSD episodes		Female PTSD episodes		Male PTSD episodes		Controls	
	mean/%	SD	mean/%	SD	mean/%	SD	mean/%	SD
**Costs (€)**	7919	15,021	6430	9130	11,021	22,654	5253	9581
**Reimbursement (€)**	7081	14,028	5795	8287	9762	21,397	5340	8986
**Length of hospital stay (days)**	12.04	15.30	10.54	10.58	15.17	21.88	7.89	8.16
**Age (in years)**	46.21	16.14	45.67	15.74	47.35	17.00	57.70	18.73
**Charlson comorbidity index**	1.29	2.40	1.32	2.40	1.24	2.39	1.93	2.75
**Female sex (%)**	68		100		0		45	
**Observations (N)**	219		148		71		34,229	

*SD* standard deviation

After risk-adjustment, PTSD was associated with additional hospitalization costs of €2311 [95%CI €1268 - €3355] per episode compared to non-PTSD patients hospitalized with the same main diagnosis, as shown in Table 2. Reimbursements rose by only €1387 [€563 - €2212], indicating that the additional costs associated with PTSD were not fully reimbursed. PTSD was associated with an additional LOS of 3.4 days [2.1–4.6 days], reflecting an additional resource use. After testing for effect modifiers for the impact of PTSD, sex proved to have an impact on the effect of PTSD on reimbursement (*p* = 0.032) and LOS (*p* = 0.041). The effect of sex on hospitalization costs was not significant (*p* = 0.091). Age and further comorbidities, operationalized trough the CCI, showed no impact on the effects of comorbid PTSD. Among male patients, PTSD was associated with additional hospitalization costs of €3846 (female: €1595), €2956 higher reimbursements (female: €686), and an extra LOS of 5.4 days (female: 2.4 days). In contrast, no gender differences were found for the control group. See Table 2 for an overview of the regression results.

**Table 2:** Additional cost, reimbursement and length of stay associated with PTSD

	All PTSD episodes vs. all controls				Male PTSD episodes vs. male controls				Female PTSD episodes vs. female controls			
	Coefficient	***p***-Value	95%CI		Coefficient	***p***-Value	95%CI		Coefficient	***p***-Value	95%CI	
***Absolute Scale***												
**Costs (€)**	2311 €	< 0.001	1268 €	3355 €	3846 €	0.004	1264 €	6428 €	1595 €	0.001	657 €	2534 €
**Reimbursement (€)**	1387 €	0.001	563 €	2212 €	2956 €	0.007	806 €	5107 €	686 €	0.053	-9 €	1381 €
**Length of stay (days)**	3.4	< 0.001	2.1	4.6	5.4	< 0.001	2.6	8.3	2.4	< 0.001	1.2	3.6
***Relative Scale***												
**Costs (€)**	36.3%	< 0.001	22.5%	50.0%	53.9%	< 0.001	25.9%	81.9%	26.9%	< 0.001	13.0%	40.8%
**Reimbursement (€)**	23.0%	< 0.001	10.8%	35.3%	43.1%	0.001	17.6%	68.6%	12.4%	0.040	0.6%	24.3%
**Length of stay (days)**	35.5%	< 0.001	24.6%	46.4%	52.1%	< 0.001	30.8%	73.4%	26.7%	< 0.001	14.9%	38.5%

**Fig. 1:**
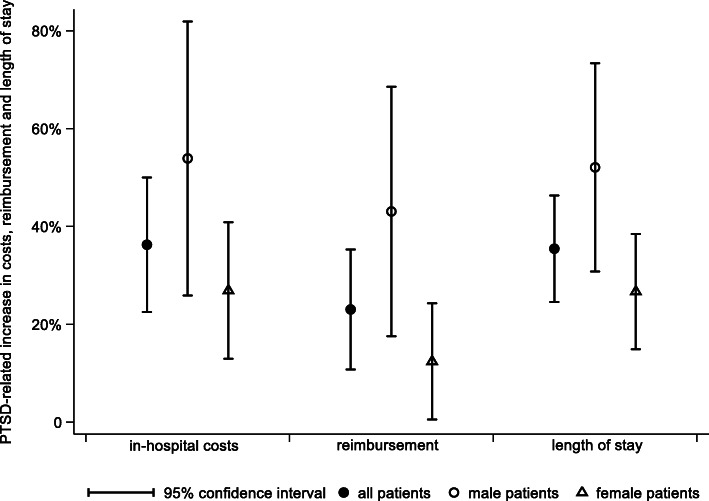
Relative PTSD-related increase in costs, reimbursement and length of stay

Figure 1 summarizes the results on the relative scale:

## Discussion

We assessed the additional hospital costs associated with PTSD in a somatic hospital using the hospital cost and reimbursement data. Inpatient episodes of patients with PTSD differed significantly from the control group. Additional costs of PTSD during hospitalization in somatic patients were €2311 on average, whereas reimbursement rose by only €1387 on average. Thus, costs were much higher and exceeded reimbursements by more than 10%, whereas in the control group reimbursements exceeded costs by 2%, indicating an inadequate reimbursement in the German reimbursement system for comorbid PTSD in somatic patients. Across all measures, male PTSD patients were less frequent than their female counterparts, but were associated with substantial higher PTSD-related excess costs, a larger cost-reimbursement gap, and a more profound PTSD-related prolongation of hospital stays than in female PTSD patients. Contrary, in psychiatric samples female gender was found to be associated with a higher LOS in previous studies [31, 32]. Generally, with an average LOS of 7.3 days in hospitals over all diagnoses, no differences between men and women can be found. However, for some diagnoses the LOS for men exceeds the LOS for women, e.g. frostbites or injuries involving multiple body parts with an LOS of 16.2 for men and 8 for women and an LOS of 13.2 for men and 8.6 for women respectively [33]. Thus, the higher LOS for men in diagnoses related to PTSD in general might be a reason for higher excess costs and reimbursement.

Prevalence of PTSD in the German population is around 2.3% in representative surveys [34–36]. Prevalence increases with age and is generally higher in women. The higher incidence of PTSD in women is mirrored in our PTSD patient group, which had a two-thirds majority of women; however patients with PTSD were actually younger than controls, despite the increase of PTSD with age reported in the population surveys [34, 37].

We further assessed if reimbursements under the G-DRG system are sufficient for patients suffering from PTSD. We found that reimbursement did increase in the presence of PTSD as comorbid disorder. However, treatment of patients with comorbid PTSD still resulted in an average lack of funding of €924€ per episode. These findings are comparable those from previous research. Hochlehnert et al. [38] found that the German reimbursement system resulted in lack of funding of €624 per hospitalization in cardiovascular inpatients with comorbid psychiatric disorders. Comparable results were observable in a more generalized study assessing all somatic primary diagnoses and all psychiatric comorbidities in a German hospital, with an average loss of -€340 [8]. In a similar study assessing associated costs of comorbid mental disorders in a medical ward, reaction to severe stress and adjustment disorders (F43) resulted in a loss of -€191 and thus are lower than our findings. However, only five patients with a comorbid F43 diagnosis could be included in that study, without further classification of exact diagnosis (e.g. F43.0 or F43.1) [9].

Physical functionality and existence of mental disorders are correlated and therefore existence of comorbid mental disorders could be seen as a proxy for severity of physical impairment [9]. For instance, PTSD has been found to be associated with premature senescence reflected in shorter leukocyte telomere length, increased pro-inflammatory markers, and prevalence of senescence-associated medical conditions, as well as higher mortality rates [39, 40]. An association with obesity has also been found [41].

Health effects of chronic sleep disturbances, often associated with PTSD [42–44] could be another factor contributing to the longer and more costly treatments in PTSD sufferers [45–47]. Part of the underlying mechanism also may be a tendency for PTSD patients to have a comorbid anxiety disorders and be more likely to experience events as traumatic [48], or experience adverse events such as panic attacks, or post-anaesthesia delirium [49, 50], which can complicate care. The physiological changes associated with the condition can also directly impact outcomes [51].

Previous researchers speculate that effects of psychiatric comorbidity on doctor-patient communication might play a role [14], and psychological or social factors associated with PTSD as either risk factors or symptoms might influence how timely and successfully a patient responds to symptoms of illness, influencing their clinical course [47, 52, 53].

### Strengths and weaknesses

Although single-center, our study describes a large all-comer patient cohort. We were able to describe the associated costs of comorbid PTSD in the somatic hospital under routine conditions. No special diagnostic or treatment of PTSD was conducted and thus the probability that we described the real inward treatment and financial situation is good. Due to the retrospective design using management data, we were able to include a high number of observations in our analysis. The high number should improve the robustness of the analysis. Nevertheless, the data used most likely did not contain information on different possible cofounders, such as information on previous diagnoses and treatments, and thus the omitted-variable bias cannot be excluded. For instance, higher health care costs of psychiatric comorbidities were associated with previous surgeries in a sample of back pain patients [7].

Furthermore of a total of 198,819 inpatient episodes with somatic diagnoses within the study period, only 0.11% had a documented comorbid PTSD. This is markedly lower than should be expected from estimates of PTSD prevalence in the population and means that there was likely a dark field of patients with existing, but undocumented PTSD. Internists are known not to detect psychological disorders in routine care [54, 55] and additionally, there is some evidence that comorbidities are not sufficiently displayed in administrative data due to different coding practices [56, 57], with comorbidities being less likely to be coded with more comorbidities occurring [57]. As we neither controlled for undetected PTSD cases nor for coding practice, it is not unlikely that we included patients with undocumented PTSD or other mental disorders in the control group. Several authors already recommend the integration of screenings in clinical interviews in routine care or training of the treating physicians in order to detect psychiatric comorbidities more systematically [54, 55]. There is already some evidence, that a structural screening for psychiatric comorbidities in somatic patient would lower the costs of inpatient care [58]. Furthermore, we did not know the treatment state of the PTSD cases and were not able to track whether and to what degree proper treatment of PTSD might improve the outcomes or cost of somatic hospitalizations.

Another limitation is, that we did not account for potential time-related biases and thus did not control for PTSD being diagnosed later in the clinical course. Additionally, we did not know the treatment state of the PTSD cases and were not able to track whether and to what degree proper treatment of PTSD might improve the outcomes or cost of somatic hospitalizations. Thus, the effects of PTSD on the associated costs might be overestimated. Furthermore, the German DRG system is in constant development, with the InEK adjusting the system in accordance with inflation and new laws every year. As major development in recent years, costs of care is additionally reimbursed to the general DRGs since 2020 [59] and thus, the overall reimbursement increased. As data used in this study were obtained between 2011 and 2014, results might differ if adjusted to new developments. However, a review by Jansen et al. [6] shows that additional costs of comorbid mental disorders are observable in different settings and reimbursement systems. Consequently, comorbid PTSD in somatic patients would most likely still be associated with additional costs in the current G-DRG system.

### Implications and future directions

In this study we found PTSD to be associated with additional health care costs in a somatic inpatient hospital. These additional health care costs were not sufficiently reimbursed by the German DRG system. If the German DRG system does not properly account for PTSD as a comorbid disorder, this constitutes a disincentive effect for hospitals to properly treat PTSD patients. This effect was already discussed in previous research [8, 21] and other authors call for an inclusion of psychiatric comorbidities into the G-DRG system [8]. Furthermore, we suggest future research regarding the impact of PTSD on the clinical course of somatic patients. If comorbid PTSD in somatic patients has an impact not only on health care costs but also the clinical outcome, a systematic diagnostic in the clinical ward and evidence-based interventions might be useful to reduce negative outcomes and health care costs.

## Conclusion

This study extends the knowledge of associated health care costs in somatic patients with comorbid mental disorders, giving a first estimate of the impact of PTSD. PTSD was associated with additional hospital costs, and reimbursement of the rendered health care services was insufficient. More evidence is needed to have a full picture of the impact of PTSD on health care costs.

## Data Availability

The datasets analyzed during the current study are not publicly available due to data protection regulations.

## Abbreviations

PTSD: Post-traumatic stress disorder
LOS: Length of stay
DRG: Diagnosis related group
ICD-10-GM: International Statistical Classification of Disease AND Related Health Problems, 10th revision, German modification
COIs: Costs of illness studies
InEK: German Institute for Hospital Reimbursement
CCI: Charlson comorbidity index
GLM: Generalized-linear model
